# Circulating MicroRNA Profiles Differ between Qi-Stagnation and Qi-Deficiency in Coronary Heart Disease Patients with Blood Stasis Syndrome

**DOI:** 10.1155/2014/926962

**Published:** 2014-12-04

**Authors:** Jincai Hou, Jun Wang, Chengren Lin, Jianhua Fu, Jianxun Ren, Lei Li, Hao Guo, Xiao Han, Jianxun Liu

**Affiliations:** ^1^Xiyuan Hospital of China Academy of Chinese Medical Sciences, 1 Xiyuan Caochang, Haidian District, Beijing 100091, China; ^2^Beijing Key Laboratory of Pharmacology of Chinese Materia Medica, Institute of Basic Medical Sciences of Xiyuan Hospital, 1 Xiyuan Caochang, Haidian District, Beijing 100091, China; ^3^Institute of Basic Theory, China Academy of Chinese Medical Sciences, 16 Dong Zhi Men Nei Nan Xiao Jie, Beijing 100700, China

## Abstract

We compared the circulating microRNA profiles of Qi-stagnation (QSB) and Qi-deficiency (QDB) in coronary heart disease (CHD) patients with blood stasis syndrome. Twenty-nine CHD patients were divided into QSB group and QDB group. The analysis was carried out through comparing their circulating microRNA profiles and the following bioinformatics analysis. The number of differential miRNAs in QDB group was much more than that in QSB group. Functional annotations of the differentially expressed miRNAs target genes in the QSB group and QDB group were, respectively, related to regulation of cellular component organization, regulation of glucose metabolic process, and so forth and protein kinase cascade, phosphate metabolic process, and so forth. KEGG pathway analysis showed that the process Qi-deficiency was associated with phagocytosis including endocytosis and mTOR signaling pathway. Specifically, pathway of cell adhesion molecules played the crucial role in the pathological process of Qi-stagnation, with a unique upregulation except for pathways associated with cancer signal. MicroRNA-gene-net analysis indicated that let-7c, miR-4487, miR-619, miR-8075, miR-6735, and miR-32-5p and miR-17-5p, miR-130a, and miR 320 family had the most important and extensive regulatory function for Qi-stagnation syndromes and Qi-deficiency syndromes, respectively. Differentially expressed miRNAs and concerned pathways suggest different molecular mechanisms that may mediate the pathological process of QSB and QDB syndromes.

## 1. Introduction

Coronary heart disease (CHD), characterized by myocardial ischemia or necrosis caused by vascular stenosis or occlusion, is one of the leading causes of deaths and hospital admissions worldwide and constitutes the leading cause of disease burden in the world according to the 2010 Global Burden of Disease Study (GBD) [1, 2]. Traditional Chinese medicine (TCM), with a 3000-year-old history that includes unique theories for aetiology and systems of diagnosis and treatment has been proven to be an effective classification method in patient stratification integrated with biomedical diagnostic method [3, 4]. TCM patterns have been used in China for thousands of years and are still playing an important role in the treatment of chronic diseases such as CHD. In TCM, the diagnosis, clinical evaluation, and treatment of CHD are based on signs and subjective symptoms according to the unique concept of “wholism.” CHD treatments are based on TCM diagnostics and syndrome differentiation which are the comprehensive responses of a certain stage in the disease process [5].

Chinese medicine holds that blood stasis syndrome is a common reason responsible for CHD in clinic of Chinese medicine due to Qi-stagnation (QSB) or Qi-deficiency (QDB). At present, many researchers consider that objective signs of blood stasis are reflected in microcirculation related to vessel and cell function, such as vascular diastolic dysfunction, abnormal platelet function, blood viscosity, and blood cell adhesion. As for QSB and QDB, there exists different biological basis and objective signs, with their respective unique characteristics of pathological changes.

MicroRNAs (miRNAs) are endogenous small RNA molecules best known for their function in posttranscriptional gene regulation. More than 60% of protein-coding genes may be targeted by miRNAs [6], mainly through translational repression and degradation of target mRNAs. MiRNAs are pivotal modulators of mammalian cardiovascular development and disease and can be steadily found in the systemic circulation of both animals and humans, where they show a remarkable stability probably due to internalization in vesicles and binding to circulating proteins and other molecules [7]. Since their levels may significantly change upon stress, circulating miRNAs have been proposed as diagnostic biomarkers in different pathologic conditions [8].

The characterization and differentiation of QSB and QDB syndromes have played an important role in the clinical practice of TCM for CHD. We speculated that miRNAs of peripheral blood have been a major parameter in discriminating the QSB and QDB syndromes and affected the appearance of the CHD patients with QSB and QDB syndromes. Therefore, we investigated the possible relationship between the syndromes as described in TCM practice and miRNAs profiles to bridge the gap between traditional syndrome diagnosis and molecular systems biology.

## 2. Materials and Methods

### 2.1. Participant Recruitment

This study was approved by the Medical Ethical Committee Xiyuan Hospital of China Academy of Chinese Medical Sciences (2011XL008-2). All 19 healthy volunteers reported no CHD and exhibited a normal syndrome as judged by TCM doctors. Delayed, written informed consent was obtained from all enrollees after they were clinically stabilized. 29 patients presenting with CHD were identified in Xiyuan Hospital of China Academy of Chinese Medical Sciences during the time between March 2012 and June 2013. Of the 29 patients screened for inclusion, 7 fulfilled study inclusion criteria for QSB group and 22 fulfilled study inclusion criteria for QDB group.

### 2.2. Sample Acquisition and Handling

10 milliliters of venous blood was obtained from ulnar vein and transferred in a sterile fashion into vacuum blood collection tubes. Samples were placed immediately on ice and then centrifuged at 4°C for 20 min at 3000 rpm to obtain the serum which was taken to the study laboratory within 10 minutes.

### 2.3. RNA Isolation and RNA Amplification

Total RNA including small RNA and miRNAs was isolated from serum samples by TRIzol reagent (Invitrogen, Canada) and purified using RNeasy Mini Kit (Qiagen, German), including a DNase digestion treatment. We ensure that the purification method retains slow molecular weight (LMW) RNA. RNA concentrations were determined by the absorbance at 260 nm and quality control standards were *A*_260_/*A*_280_ = 1.8–2.1, using NanoDrop 2000 (Thermo, America).

### 2.4. Affymetrix miRNA Microarray

RNA was labeled using FlashTag Biotin HSR labeling kit as the manufacturer's instructions (Genisphere, America). Labeled RNA was hybridized to GeneChip microRNA 3.0 array (Affymetrix, America) according to the user manuals. Affymetrix Expression Console Software (version 1.3.1) was used for microarray analysis, including data normalization, summarization, and quality control assessment. Median-centric normalization was used for the custom microRNA oligonucleotide chips. Affymetrix chips were normalized using the robust multichip analysis (RMA) procedure. Differentially expressed miRNAs were identified based on RVM *t*-test analysis. Differentially expressed miRNAs with at least 1.5-fold change in either direction with *P* < 0.05 were considered to be up- or downregulated.

### 2.5. Quantitative Real Time RT-PCR Validation

The cDNA was subjected to real time quantitative PCR with defined primers and Power SYBR Green PCR Master Mix (Applied Biosystems, Foster City, CA, USA). The data were analyzed using the ABI 7000 system SDS software (Applied Biosystems, Foster City, CA, USA). All the experiments were performed in duplicate and relative expression levels of these microRNAs were determined by the 2^−ΔΔCt^ method.

### 2.6. Gene Ontology (GO) Analysis

Gene ontology analysis has been carried out using DAVID and GSEA. ID of differential expressed genes was uploaded to the DAVID database and the analysis was performed based on their respective molecular function, biological process, or cellular component. Functional categories were enriched within genes that were differentially expressed between the QSB and QDB groups. Top ten GO functional categories that genes mainly involved in were selected for specific analysis.

### 2.7. Pathway Analysis

Normalised signal intensities from each experimental condition for the differentially expressed genes were uploaded to the KEGG database which is to collect pathway maps that computerize the network information of molecular interaction. KEGG analysis (KEGG data version: Release 70.1, June 1, 2014) is used for discovering the relation that is not easily visible from the changes of individual genes. Pathways that had significant changes of *P* < 0.05 and fold change > 1.5 were identified for further analysis.

### 2.8. MicroRNA-Gene-Net Analysis

To build a miRNA-gene-network, the relationship between miRNAs and genes was counted by their differential expression values and according to their interactions in the Sanger (Targetscan&miRanda) miRNA database. TargetScan 6.2 (http://www.targetscan.org/) in conjunction with the miRanda version August 2010 Release (available at: http://www.microrna.org/) was used to predict the targets of the miRNAs. The circles represented target genes and the squares represented miRNAs. The relationships between miRNAs and target genes were represented by edges. The center of the network was represented by degree which is the contribution of one miRNA to the genes around or the contribution of one gene to the miRNAs around [9]. The key miRNA and gene in the network always have the biggest degrees.

## 3. Results

### 3.1. Patient Demographic

As shown in Table 1, the age of QSB group (65.43 ± 9.55) had no significant difference compared with QDB (66.55 ± 11.74). Most participants presenting with QSB and QDB syndromes in our cohort were uniformly male, with the percentage of 71.43% in QSB group and 63.64% in QDB group. 57.1% of QSB patients and 72.7% of QDB patients had complications including hypertension, diabetes, and hyperlipidemia. The majority of participants in both QSB and QDB groups had a history of hypertension (60% and 93.7%, resp.) and a minority had a history of diabetes or hyperlipidemia (40% and 16.3%, resp.).

### 3.2. Identification of miRNAs from QSB and QDB Syndromes by miRNA Array

**Figure 1:**
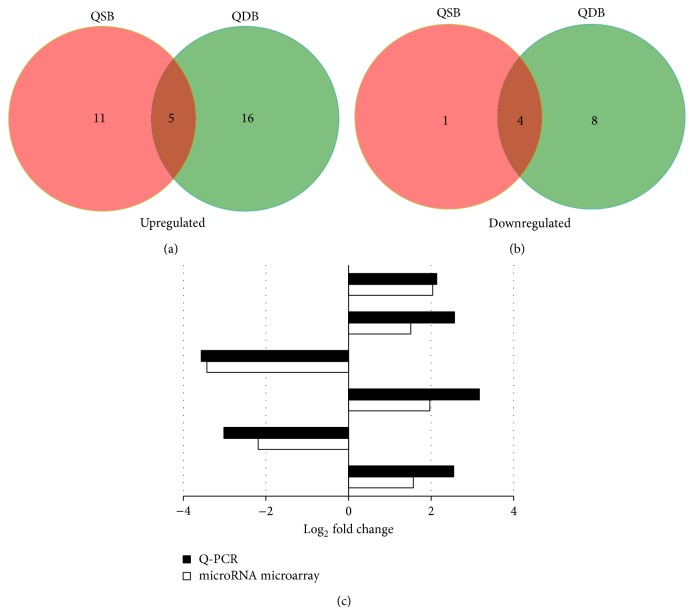
(a) Section of upregulated differentially miRNAs overlapping and nonoverlapping in QSB and QDB groups. (b) Section of downregulated differentially miRNAs overlapping and nonoverlapping in QSB and QDB groups. (c) Real time qRT-PCR validation of the microarray results: microarray results compared to qRT-PCR results relative to the OC. The obvious upregulated or downregulated miRNAs in QSB and QDB groups from microarray data (FDR *P* < 0.05; fold change > 1.5) were randomly selected. Expression changes are depicted as log_2_ fold change (*x*-axis). miRNAs symbols are shown on the other side of the column.

MiRNAs that are differentially expressed between QSB and QDB groups which are expected to contribute to functional differences among the CHD patients. Therefore, we identified miRNAs that are differentially expressed (*P* < 0.05) between QSB and QDB by microarrays. Compared with the healthy patients, we detected 21 differentially expressed miRNAs, with 16 (76.2%) upregulated miRNAs and 5 (23.8%) downregulated miRNAs. 33 differentially expressed miRNAs were found in QDB group, with 21 (63.7%) upregulated miRNAs and 12 (36.3%) downregulated miRNAs.  Figure 1 displayed the differentially expressed miRNAs overlapping and nonoverlapping in QSB and QDB groups, which showed that 5 upregulated (Figure 1(a)) and 4 downregulated (Figure 1(b)) differentially expressed miRNAs overlapping among the 2 groups.

### 3.3. qRT-PCR Validation

To determine that the microarray-based miRNAs detections were reliable for the determination of miRNAs expression patterns in the serum, we performed SYBR green-based real time quantitative PCR on upregulated or downregulated miRNAs in the QSB and QDB groups from microarray data (FDR *P* < 0.05; fold change > 1.5). All randomly selected miRNAs were in agreement with the microarray analyses in terms of the direction of the observed differential expression (Figure 1(c)). Specifically, the observed log_2_ fold changes with qRT-PCR of the upregulated miRNAs (miR-451a, miR-23b, miR-455, and miR-32) were the same positive tendency as obtained with the microarray data. Equally, the observed log_2_ fold changes with qRT-PCR of the down-regulated miRNAs (miR-320c and miR-619) were the same negative tendency as obtained with the microarray data.

### 3.4. Gene Ontology (GO) Analysis of the miRNAs Target Genes

**Figure 2:**
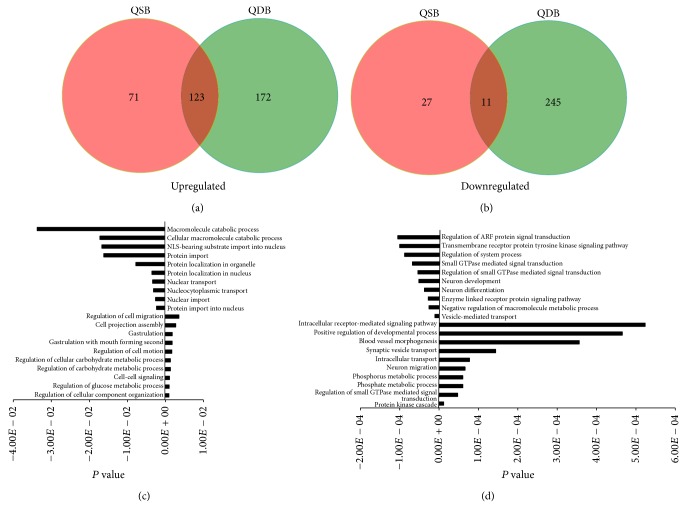
(a) Section of overlapping and nonoverlapping upregulated functional annotations from the differentially expressed miRNAs target genes. (b) Section of overlapping and nonoverlapping downregulated functional annotations from the differentially expressed miRNAs target genes. (c) Bar graphs showed the most obvious upregulated and downregulated functional annotations of QSB group. (d) Bar graphs showed the most obvious upregulated and downregulated functional annotations of QDB group. Expression changes are depicted as *P* value (*x*-axis). GO symbols are shown on the other side of the column.

In an effort to better understand molecular function or biological process difference between circulating miRNA profiles in patients with QSB and QDB syndromes, we compared the gene ontology of miRNAs target genes. Venn diagram displayed the overlapping and nonoverlapping functional annotations in QSB and QDB groups, which showed that 123 upregulated (Figure 2(a)) and 11 downregulated (Figure 2(b)) differentially functional annotations overlapped among the QSB and QDB groups. According to the GO analysis, the obvious upregulated and downregulated functional annotations of the differentially expressed miRNAs target genes in the QSB group (Figure 2(c)) were related to regulation of cellular component organization, regulation of glucose metabolic process, cell-cell signaling, and so forth and protein import into nucleus, nuclear import, nucleocytoplasmic transport, and so forth, respectively. Based on the function of differentially expressed miRNAs target genes in the QDB group (Figure 2(d)), the obvious upregulated and downregulated functional annotations were mainly related to protein kinase cascade, regulation of small GTPase mediated signal transduction, phosphate metabolic process, and so forth and vesicle-mediated transport, negative regulation of macromolecule metabolic process, enzyme linked receptor protein signaling pathway, and so forth, respectively.

### 3.5. Pathway Analysis of miRNAs Target Genes

**Figure 3:**
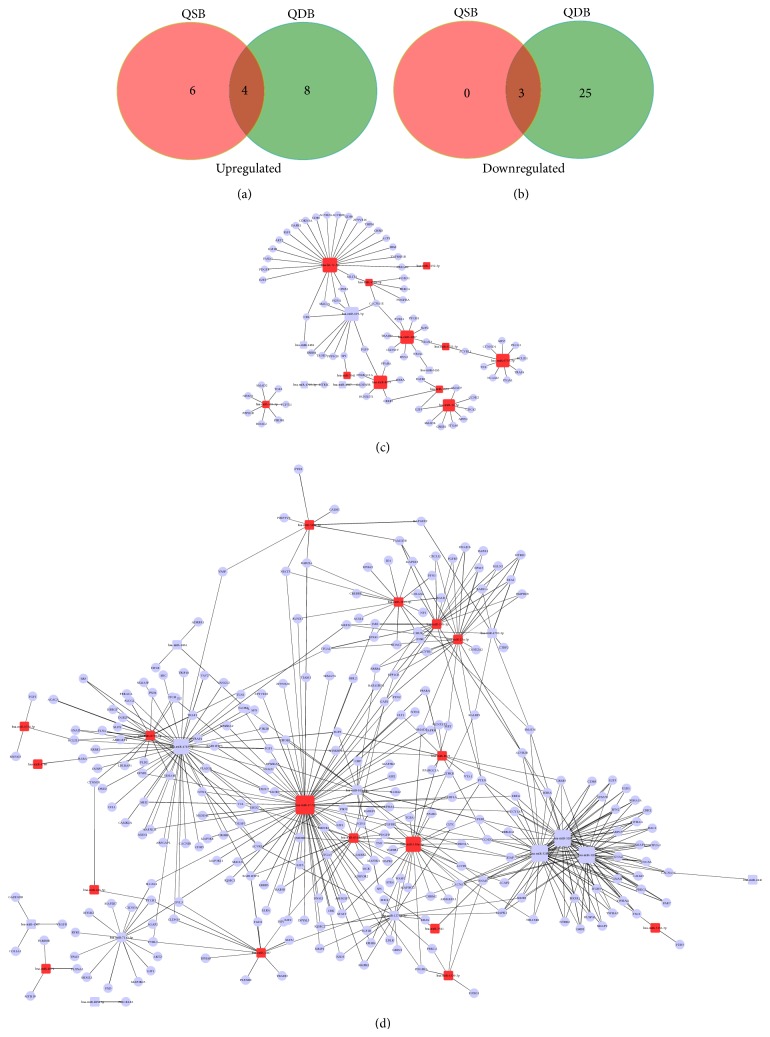
(a) Venn diagram showed overlapping and nonoverlapping upregulated pathways in QSB and QDB groups. (b) Venn diagram showed overlapping and nonoverlapping downregulated pathways in QSB and QDB groups. (c) The microRNA-gene-network diagram of QSB. (d) The microRNA-gene-network diagram of QDB. Red squares represent upregulated miRNAs and the blue squares represent downregulated miRNAs. Blue rounds represent the target genes of their connected miRNAs. Black lines represent the regulatory relation between miRNAs and their target genes.

Based on the KEGG database, 4 upregulated (Figure 3(a)) and 3 downregulated (Figure 3(b)) overlapping pathways were identified in QSB group and QDB group including pathways in cancer, TGF-beta signaling pathway, and calcium signaling pathway. Moreover, 6 pathways were remarkably upregulated in the QSB group, and 8 and 25 pathways were significantly downregulated in QDB group.  Table 2 demonstrated that cell adhesion molecules (CAMs) played the crucial role in the pathological process of Qi-stagnation, with a significant and unique upregulation besides the rest cancer related pathways.  Table 3 displayed that Qi-deficiency was associated with endocytosis and mTOR signaling pathway which is involved in the process of autophagy.

### 3.6. MicroRNA-Gene-Net Analysis

Gene regulatory networks composed of miRNAs and their target mRNAs are expected to be important for the biological function of the specific syndromes. Therefore, we carried out pathway analysis for miRNAs that were differentially expressed between QSB and QDB and that belonged to miRNA clusters or families. The network diagram of QSB group showed the core miRNAs and their target genes including let-7c, miR-4487, miR-619, miR-8075, miR-6735, and miR-32-5p (Figure 3(c)). As for QDB group, miR-17-5p, miR-130a, miR-320a, miR-320b, and miR-320c displayed the important role in the network (Figure 3(d)).

## 4. Discussion

In the long history of traditional clinical practice in China and other Eastern countries, TCM practitioners have typically classified patients of the same disease into subgroups as different syndromes from a holistic perspective on patients' overall status [10]. Qi-blood theory, a complicated and intricate theory, is one of the basic theories of TCM. Qi is used to describe the refined nutritious substances constituting the human body and maintaining life activities. The concept of blood in TCM is also used to describe body's functions and is always regarded the same as the blood in western medicine in most conditions, with a clearer definition than Qi [11]. Qi-blood theory is widely used to in the diagnosis and treatment for the chronic diseases such as CHD [12]. However, Qi-stagnation (QSB) and Qi-deficiency (QDB) symptoms in CHD patients are subjective and difficult to evaluate objectively. We hypothesized that microRNAs from QSB and QDB appearances may reflect characteristics of the syndromes, which is associated with the status of the Qi-blood.

According to the analysis of the differential miRNAs profiles, significantly upregulated or downregulated miRNAs are overlapped in the QSB and QDB groups, such as miR-451a, miR-3152, and miR-4487. This indicates that the parts of mechanism for QSB and QDB groups were similar and may be the basis of QSB and QDB syndrome. Based on the upregulated or downregulated value in QSB, the most significant upregulated miRNA is miR-7641 which can significantly suppress CXCL1, a member of the CXC chemokine family that promotes neovascularization by binding G-protein coupled receptors and is related to endothelial cells biogenesis such as angiogenesis [13]. Consequently, the upregulated miR-7641 in QSB patients suggests an abnormal endothelial cells function which possibly results for the inhibition of angiogenesis. The most significant downregulated miRNA in QSB group is miR-4484 which differentially expressed between macrophages infected with virulent and avirulent mycobacterium tuberculosis [14]. In addition to endothelial cells function, the macrophage dysfunction may be another biological basic of QSB syndrome. According to the log_2_ fold change value in QDB group, the most obvious downregulated miRNA is miR-320c. It was evidenced that downregulation of miR-320 induced the overexpression of proinflammatory cytokines through the modulation of ERK/NF-*κ*B pathways [15]. Therefore, in the process of QDB, downregulated miR-320 may contribute to the proinflammatory cytokines generation.

Based on the results of GO analysis, the functions of most differentially regulated genes in QSB were related to the regulation of cellular component organization, protein import into nucleus, glucose metabolic process, and so forth, which indicated that the mechanism for QSB syndrome might relate to the dysfunction of the above biological process. As for the QDB, the functions of most differentially regulated genes were related to protein kinase cascade, regulation of small GTPase mediated signal transduction, vesicle-mediated transport, and so forth, suggesting that QSB syndrome might relate to the dysfunction of the above biological process.

According to the KEGG analysis, 6 pathways were related to the QSB, 5 of which were related to the cancer concerned pathways. The only pathway that did not belong to the cancer concerned pathway was cell adhesion molecules (CAMs). Cell-cell and cell-matrix interactions are mediated through several different families of CAMs including the selectins, the integrins, the cadherins, and the immunoglobulins. CAMs play a very significant and critical role in both heart development and different pathophysiological heart disease [16]. Blood stasis syndrome has a certain degree of high coagulation state. On this pathological basis, cell adhesion molecules were more significant in the QSB than that in QDB which indicated that CAMs play more important role in QSB. We assumed that several members in the CAMs superfamilies and in particular the integrin family will serve as diagnostic strategies with QSB. The number of pathway with obvious change in QDB group is more than QSB group, parts of which are associated with phagocytosis such as endocytosis, mTOR signaling pathway which is involved in the process of autophagy. All types of cells in the body use the endocytosis process to communicate with the biological environments. This process is an energy-dependent process through which cells internalize ions and biomolecules [17]. In addition, it is evidenced that the cellular rate of nutrient or ion uptake (e.g., glucose, Fe^3+^, and K^+^) or efflux (e.g., Na^+^) is governed by a complement of membrane transporters and receptors that show dynamic localization at both the plasma membrane and the defined intracellular membrane compartments [18]. Some research showed that endocytosis was upregulated in border zone cardiomyocytes, and inhibition of endocytosis might be an effective approach to prevent export of injury signals from the myocardial infarct size [19]. In considering endocytosis as the most obvious upregulated pathway in QDB, we speculate that endocytosis process consumes excessive the above mentioned nutrients so that the body appears a series of energy deficiency signs, as so-called Qi-deficiency. mTOR signaling pathway is one of regulatory molecules that negatively controls autophagy [20–22]. Consequently, the downregulated mTOR signaling pathway in QDB can activate autophagy which is evidenced to be harmful for myocardial ischemia reperfusion [23, 24]. The downregulated mTOR signaling pathway in CHD patients with QDB syndrome results in the activation of autophagy which could increase myocardial cell apoptosis. Therefore, the autophagy may be another biological characteristic of QDB syndrome different from QSB syndrome.

The construction of microRNA-gene-network in QSB group demonstrates that let-7c, miR-4487, miR-619, miR-8075, miR-6735, and miR-32-5p are located in the center of diagram, suggesting that the abnormal miRNAs may have the most important and extensive regulatory function for Qi-stagnation. The construction of microRNA-gene-network in QDB displays that miR-17-5p, miR-130a, miR-320a, miR-320b, and miR-320c are located in the center of diagram, suggesting that the abnormal miRNAs may have the most important and extensive regulatory function for Qi-deficiency. Further, we found that though miR-4487, miR-619, and miR-8075 were the overlapping miRNAs in both QSB and QDB syndromes, their regulatory function seemed more wide and involved in more regulatory networks in QSB syndromes than that in QDB syndromes. Of note, miR-320 family including miR-320a, miR-320b, and miR-320c regulates QDB syndromes predominantly. MiR-320 has been found to have widespread biological effects as it regulates multiple important molecules. Its potential targets include ET-1, ERK1, VEGF, and FN (http://www.microrna.org/). The biologic actions of miR-320 involve carcinogenesis, development, and ischemia reperfusion injury [25, 26]. Combined with Qi-deficiency characteristic, we consider that miR-320 family plays a crucial role in various performances with insufficient energy and nutrient.

In this paper, we compared molecular networks of two syndromes of CDH patients with blood stasis and analyzed their common and different microRNAs mechanisms. We achieve a better understanding of the biological characteristics of syndrome differentiation of CHD which is beneficial to explore different therapies to enhance the effectiveness and pertinence of treatment.

## Figures and Tables

**Table 1:** Demographic characteristics of CHD patients with QSB and QDB syndrome.

	QSB (*N* = 7)	QDB (*N* = 22)
Demographics:		
Mean age (years)	65.43 ± 9.5	66.55 ± 11.7
Sex (Male)	4	8
Heart rate (BPM)	83.2 ± 6.8	77.8 ± 10.7
Blood pressure (SBP/DBP)	144 ± 12.1/90.6 ± 11.3	140.5 ± 5.23/81.8 ± 4.1
Body mass index (kg/m^2^), mean ± SE	27.9 ± 0.6	26.2 ± 0.85
Comorbid conditions:		
Hypertension	3 (42)	15 (68)
Diabetes mellitus	1 (14)	4 (18)
Hyperlipidemia	1 (14)	4 (18)
Current smoking	4 (57)	13 (59)
Alcohol	2 (28)	6 (27)
Prehospital medications:		
Statins	5 (71)	20 (90)
Beta blocker	1 (14)	5 (22)
Angiotensin-converting enzyme inhibitor/angiotensin receptor blocker	2 (28)	8 (36)
Calcium channel blocker	2 (28)	6 (27)
Aspirin	3 (43)	9 (41)
Nitroglycerin	3 (43)	7 (32)

**Table 2:** Upregulated pathways in patients with QSB syndrome.

Path_ID	Path_name	Count	%	*P* value	FDR
hsa05215	Prostate cancer	16	1.394943	2.63*E* − 04	0.020708
hsa05214	Glioma	12	1.046207	0.00128	0.065618
hsa05218	Melanoma	12	1.046207	0.003441	0.128043
hsa04514	Cell adhesion molecules (CAMs)	17	1.482127	0.006066	0.175925
hsa05222	Small cell lung cancer	11	0.959024	0.030494	0.42138
hsa05223	Non-small cell lung cancer	8	0.697472	0.043989	0.510943

**(a):** 

Path_ID	Path_name	Count	%	*P* value	FDR
hsa04144	Endocytosis	41	0.245627	5.65*E* − 08	9.66*E* − 06
hsa04360	Axon guidance	28	0.167745	1.84*E* − 05	0.00157
hsa05212	Pancreatic cancer	15	0.089863	0.003962	0.126948
hsa04010	MAPK signaling pathway	36	0.215672	0.01223	0.259629
hsa05220	Chronic myeloid leukemia	14	0.083873	0.014293	0.239301
hsa04070	Phosphatidylinositol signaling system	13	0.077882	0.029505	0.400777
hsa04810	Regulation of actin cytoskeleton	28	0.167745	0.041864	0.485632
hsa04012	ErbB signaling pathway	14	0.083873	0.043572	0.469973

**(b):** 

Path_ID	Path_name	Count	%	*P* value	FDR
hsa04012	ErbB signaling pathway	18	1.880878	1.15*E* − 05	0.001671
hsa05214	Glioma	14	1.462905	6.91*E* − 05	0.00503
hsa04910	Insulin signaling pathway	21	2.194357	1.33*E* − 04	0.006429
hsa04510	Focal adhesion	27	2.821317	1.39*E* − 04	0.005062
hsa04730	Long-term depression	13	1.358412	6.98*E* − 04	0.016838
hsa05220	Chronic myeloid leukemia	13	1.358412	0.001496	0.030734
hsa04720	Long-term potentiation	12	1.253918	0.002133	0.038212
hsa04722	Neurotrophin signaling pathway	17	1.776385	0.002769	0.039679
hsa04150	mTOR signaling pathway	10	1.044932	0.003329	0.043298
hsa05212	Pancreatic cancer	12	1.253918	0.003394	0.04052
